# The Chemistry of the Ketogenic Diet: Updates and Opportunities in Organic Synthesis

**DOI:** 10.3390/ijms22105230

**Published:** 2021-05-15

**Authors:** Michael Scott Williams, Edward Turos

**Affiliations:** Department of Chemistry, University of South Florida, Tampa, FL 33620, USA

**Keywords:** ketogenic diet, ketosis, keto, epilepsy, exogenous ketone supplements, acetoacetate, β-hydroxybutyrate

## Abstract

The high-fat, low-carbohydrate (ketogenic) diet has grown in popularity in the last decade as a weight loss tool. Research into the diet’s effects on the body have revealed a variety of other health benefits. The use of exogenous ketone supplements to confer the benefits of the diet without strict adherence to it represents an exciting new area of focus. Synthetic ketogenic compounds are of particular interest that has received very little emphasis and is an untapped area of focus for chemical synthesis. In this review, we summarize the chemical basis for ketogenicity and opportunities for further advancement of the field.

## 1. Introduction

The ketogenic diet, a high-fat, low-carbohydrate diet, has a long history of use beginning primarily as a treatment option for epilepsy [1,2]. However, it is only in the last few decades that the diet has been popularized among the general public. Renewed clinical interest in the diet and its emerging popularity as a weight-loss tool have led to a larger scope of research into the diet’s effects on the body and the discovery of a broad range of physical, biochemical, and cosmetic benefits.

Even with expanding research interest in the ketogenic diet, most prior reported studies have been done in clinical settings or in biology labs [3,4]. Emerging research shows that certain ketogenic supplements can confer some of the benefits of the ketogenic diet while following a normal diet [5,6]. Difficulty in adherence to the ketogenic diet is often cited as a reason why it is abandoned due to limited food options. If these obstacles can be overcome, a wide array of possibilities may open up. Yet, very little work has been done so far in expanding the options to do this, particularly from the chemistry side. This review will cover the history and health-related aspects of the ketogenic diet and its biochemical basis as well as highlight some exciting opportunities in organic synthesis to devise routes to potential new ketogenic compounds as components or supplements of the ketogenic diet.

## 2. History of the Ketogenic Diet

Dietary fasting has been a common societal practice in various religions for thousands of years, and as a treatment for epilepsy for over a century. Much of the early reported information is based on the personal experience of cultists and physicians instead of scientifically motivated clinical trials. In 1911, two French physicians Guelpa and Marie published a report detailing their use of fasting to treat epileptic seizures, in which six of their 26 patients showed a reduction in the severity of their seizures or the rate of seizure occurrence [7]. No further details were provided. At the American Medical Association convention in 1921, Geyelin presented a report of a controlled study involving 36 epileptic patients who used fasting as a treatment plan [8]. The results showed that 80% of these patients experienced a decrease in the number of seizures. Despite this promising outcome, fasting saw sporadic use among physicians to try to control epileptic seizures, but no formal clinical trials were done and its use as a long-term treatment method was obviously not viable.

**Figure 1:**
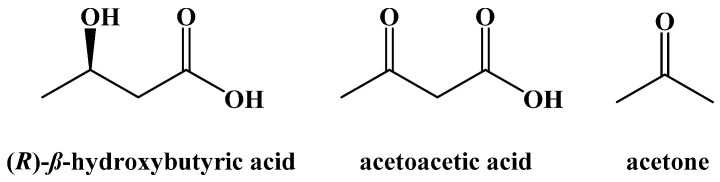
The Ketone Bodies.

Around the same time, research into the use of diet modification to control diabetes mellitus was developing. The difference between type 1 and type 2 diabetes was not understood at that time and so there was no distinction made. Newburgh and Marsh reported the use of a high-fat, low-carbohydrate diet to manage diabetics in 1920 [9,10,11]. In 1921, Woodyatt published a review article on the modification of diet to control diabetes mellitus based on his own research and that of Philip Shaffer, who sought to study the relationship between acetoacetic acid and glucose in vitro [12,13]. It had long been known that diabetic patients often had an increased concentration of acetoacetic acid, (R)-β-hydroxybutyric acid, and acetone in their blood (ketonemia) and urine (ketourea) based on research done by Gerhardt in 1865 [14]. These three compounds are collectively known as the ketone bodies (Figure 1).

This increase in blood concentrations of the ketone bodies had been shown by Geelmudyen in 1897 to be caused by an increase in the metabolism of fatty acids, due to diabetic patients’ inability to metabolize glucose at a rate fast enough to meet caloric needs [15]. This can be dangerous for those with diabetes because unchecked ketonemia can lead to a state we now know as diabetic ketoacidosis, where excess ketone bodies acidify the blood, leading to nausea, weakness, and even death. Woodyatt suggested a diet based on Newburgh and Marsh’s research where the amount of carbohydrates consumed did not exceed the quantity the body could metabolize, and to supplement the diet with fats. This prevented the buildup of sugar in the blood (hyperglycemia), one of the causes of ketonemia. Woodyatt believed that over time, resting the pancreas would increase the body’s capability to metabolize carbohydrates and a normal diet could be resumed. The discovery of insulin in 1921 and its use to treat diabetes mellitus starting in 1922 decreased the need for other treatment options, but the interest in high-fat, low-carbohydrate diets continued to grow [16].

Later that same year, Wilder published a report based on Geyelin’s use of fasting as a treatment for epilepsy and suggested that it may be due to the patients’ ketonemia [17]. Referencing Shaffer’s work, Wilder suggested inducing ketonemia not through fasting, but through a high-fat, low-carbohydrate diet he referred to as the “ketogenic diet”. Peterman, one of his colleagues, formulated the optimized amounts of macronutrients for the diet, as being no more than 15 g of carbohydrates a day, 1 g of protein per kilogram of body weight, and the remaining caloric deficit made up of fats. This is nearly identical to the modern ketogenic diet, a 4:1 ratio of fats to carbohydrates and protein with a maximum allowance of 50 g of carbohydrates per day. Wilder released a report shortly after this on three of his patients who saw a sharp decrease in the rate of seizures [18].

Further studies over the next few years corroborated the use of the ketogenic diet as a treatment for epilepsy [19]. Peterman reported his own observations in 1924 across two studies on the use of the ketogenic diet compared to a variety of other treatments. In the first, nine out of 13 patients using only the ketogenic diet as treatment for their epilepsy were free of seizures for up to a year [1]. In the second study, 19 out of 37 were seizure-free for up to two years [20]. In 1927, Talbot reported a study of 200 children where the ketogenic diet provided complete symptomatic relief among a third and partial improvement to three quarters [21]. Helmholz reported the use of the diet across 144 epileptic patients. Forty-six patients were seizure-free, and 34 saw a decrease in the rates of seizure occurrence [22].

**Figure 2:**
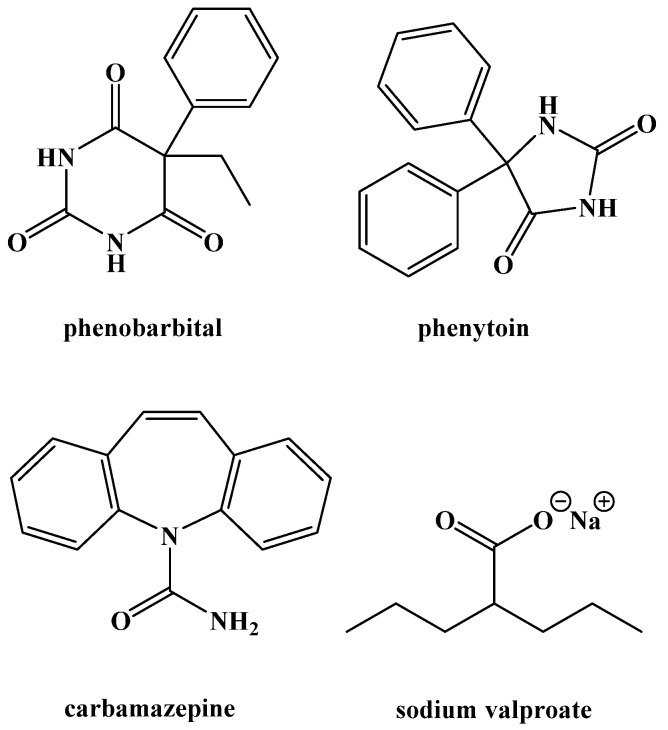
Common Anticonvulsant Medications.

The use of medication to treat epilepsy was gaining popularity around this time as well (Figure 2). Phenobarbital was brought to market as an anti-convulsant in 1912 and was one of the treatment options Peterman compared to the ketogenic diet [1,23]. Phenytoin was synthesized in 1908 and shown to be useful for preventing seizures in 1936 [24]. Carbamazepine and sodium valproate were both brought to market in 1962 [25,26]. All are still used and are currently on the World Health Organization Model List of Essential Medicines. The emergence of novel anticonvulsant medications caused the ketogenic diet to decrease in popularity as a treatment option, possibly because it was thought that medicinal options would eliminate behavioral changes altogether [27]. Nonetheless, the ketogenic diet never completely disappeared. The difficulty in developing a widely accepted medicinal treatment protocol to control epileptic seizures led to its sporadic use.

Johns Hopkins Hospital in particular seemed to use the diet for epilepsy treatment more often than most health care centers, publishing reviews and reports on its efficacy in the 1980s and 1990s [27,28,29]. The cause of the diet’s subsequent upswing in popularity is at least partially due to the efforts of Jim Abrahams, a film director whose son Charlie’s seizures were treated successfully at Johns Hopkins using the ketogenic diet. His son’s story was documented on Dateline NBC in 1994 and he started a foundation called the Charlie Foundation to spread awareness and fund research into the ketogenic diet [30]. It is likely because of this increase in publicity that popularity and interest in the diet grew, leading to a surge of research in the past twenty-five years [31]. This research has helped to expand the benefits of the ketogenic diet beyond its use as a treatment for epilepsy [32].

While early use of the ketogenic diet to treat epilepsy was done without a full understanding of the biochemistry through which ketone bodies are generated and utilized, research over the past century has further elucidated these pathways.

## 3. Competing Energy Sources in the Body

**Figure 3:**
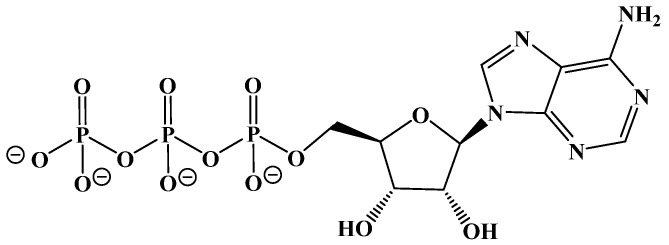
Adenosine Triphosphate (ATP).

Energy for the body comes from the digestion and metabolic breakdown of macronutrients (fats, carbohydrates, and protein) in the diet. The body prioritizes carbohydrate metabolism due to their availability from the diet and from stored glycogen in the liver and in muscles, chemically converting them through hydrolysis to glucose, which is oxidatively broken down through glycolysis to S-acetyl coenzyme A (or S-acetyl CoA). S-Acetyl CoA is needed to fuel the Krebs cycle, which completes the conversion of upstream carbon sources to carbon dioxide and adenosine triphosphate (ATP), the primary source of energy in cells (Figure 3).

**Figure 4:**
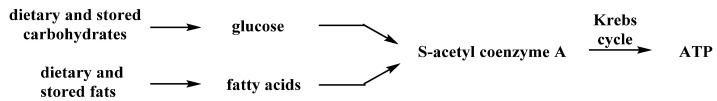
Metabolic Sources of S-Acetyl CoA.

Fats can likewise be broken down hydrolytically to fatty acids that metabolically produce S-acetyl CoA for the Krebs cycle. This occurs through a repetitive β-oxidation process in the mitochondrion of liver cells (mostly). Normally, these two pathways to S-acetyl CoA are complementary and carefully regulated metabolically. However, when pushed beyond this regulation by dietary consumption or by the body’s immediate energy needs, the pathways can become competitive. Indeed, by limiting the availability of carbohydrates in the diet and the accumulation of stored glucose (glycogen reserves), the body can switch to the use of dietary or stored fats to produce the S-acetyl CoA needed for the Krebs cycle (Figure 4).

## 4. Dietary Fats

**Figure 5:**
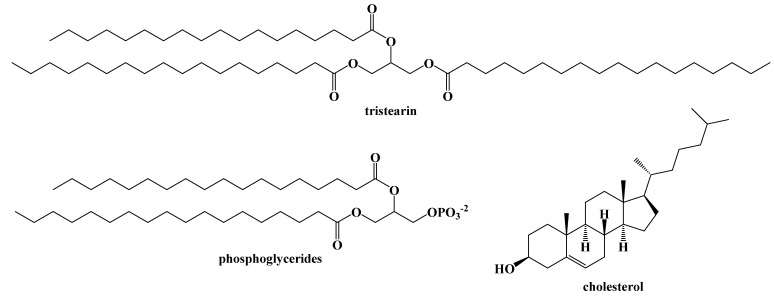
Examples of Common Dietary Fats.

While the ratio of macronutrients in the ketogenic diet has remained relatively unchanged since its development in 1921, there is some variety within the dietary fats we consume. Fats are lipophilic, largely water-insoluble substances that can be extracted from living cells with common organic solvents, such as hexane or diethyl ether, and depending on their structures, can appear as oils, semi-solids, or greasy waxes at room temperature. Collectively, these organic-soluble compounds can be referred to commonly as fats, and include a wide range of structural types that include long chain ester derivatives, such as triacylglycerides, branched chain compounds comprised of a wide assortment of terpenes and terpenoids, such as the steroids (e.g., cholesterol) and their fatty esters. Figure 5 illustrates some examples of common dietary lipids.

**Figure 6:**
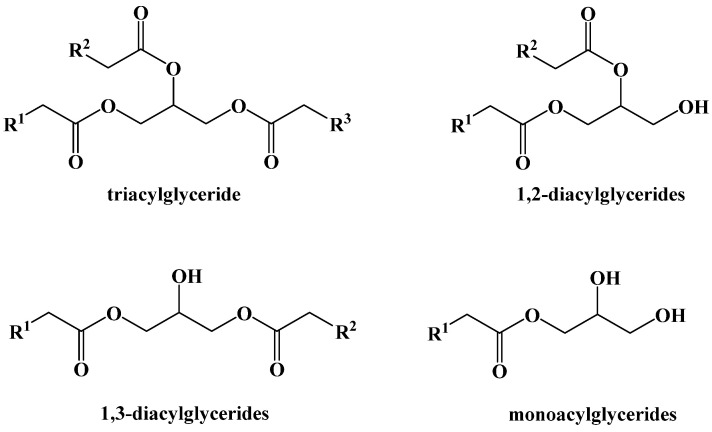
Dietary Acylglyceride Fats.

Dietary fats are primarily triacylglycerides (triglycerides), a glycerol backbone bound to three carboxylic acids with long aliphatic chains (fatty acids) via ester bonds. Monoacylglycerides and diacylglycerides are also naturally occurring, but not as abundant in the diet (Figure 6).

The triacylglycerides found in the diet are composed of three long-chain fatty acids (between 14 and 22 carbons) and may be identical or different in the carbon chain length or in the presence of unsaturation or oxygenation in the chain [33]. The number of alkenes in the chain can vary as well, from being saturated, monounsaturated, or polyunsaturated. The alkene geometry can likewise be either E or Z, although nearly all examples in nature are Z. The length of the carbon chain, and the location or the type of alkene(s) within the fatty ester chain, largely determine the physical and to some extent the biochemical properties of the triglyceride, with fully saturated chains or those having E-alkenes being more conformationally-rigid and leading to compounds with higher melting points. Plants and animals that live in cold environments produce a greater abundance of triglycerides and other fatty lipids that remain fluid at low temperatures, depending on the required function and physical state.

**Figure 7:**
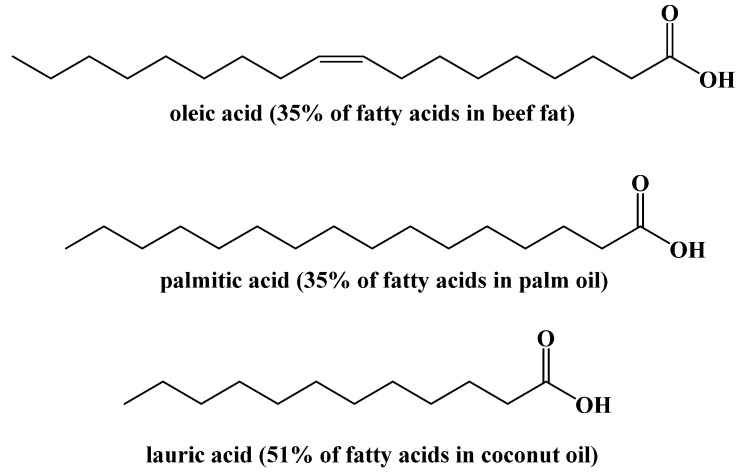
Major Fatty Acids in Common Foods.

The fatty acid concentrations in triacylglycerides also vary greatly based on the source of fat. The concentrations of specific fatty acids in different sources of fat can be analyzed by cleaving the fatty acids from triacylglycerides and converting them into their ester forms. Using these methods, the primary fatty acids in different fats has been studied (Figure 7) [33,34].

These concentrations can also be affected by environmental factors within specific dietary fat sources [35].

The long alkyl chains found in most dietary fats are useful biofuels for cells, as their carbons are in a higher reduction state (typically -2 or -3) than those in carbohydrates (around 0). Consequently, fats store about twice the amount of potential energy within their structures, which is released when converted in cells to CO_2_.

## 5. Biochemistry of the Ketogenic Diet

**Figure 8:**
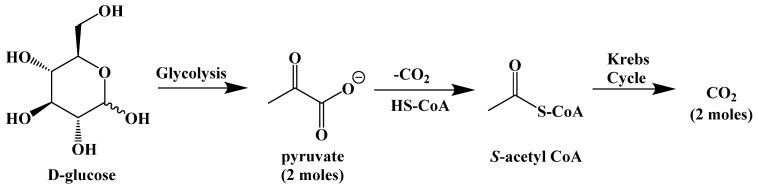
Metabolism of Glucose.

The biochemistry behind the ketogenic diet is relatively simple. The ketogenic diet attempts to emulate the body’s response to starvation or fasting by eliminating carbohydrates as the provider of S-acetyl CoA, and thus the body´s source of energy. When carbohydrates are unrestricted in the diet, the glucose present in cells or in the blood is enzymatically converted to pyruvate through the glycolytic pathway (Figure 8). 

The glycolysis pathway generates energy in the form of adenosine triphosphate (ATP). The pyruvate molecule is then converted to S-acetyl CoA to be used as fuel for the Krebs cycle, the body’s primary process for generating energy in the form of ATP (and its direct link to the mitochondrial electron-transport chain).

When carbohydrates are abundantly available in the food we eat, the increase in blood glucose concentration signals the pancreas to secrete insulin, a protein hormone that stimulates the cellular absorption of glucose from the blood. Insulin-dependent glucose uptake is largely responsible for excess glucose storage in the liver and skeletal muscle as glycogen, and for the conversion of glucose to glycerol (along with glycerol from the hydrolysis of triglycerides) to be used in the biosynthesis of triacylglycerides that end up being stored in adipose tissue [36]. As a result of these processes, blood glucose concentration is maintained at a steady state of around 100–140 mg/dL of blood, and upon the burning of glucose through the Krebs cycle, ultimately decreases to a point where glucagon (another protein hormone) is released by the pancreas to orchestrate the break-down of stored glycogen to release more glucose into the blood. These glycogen stores typically last for about a day. When more carbohydrates are consumed, more insulin is released to accelerate the uptake of glucose to replenish glycogen stores, and the process repeats itself [37]. An average 180-pound man typically stores around one pound of glycogen in the liver (giving about a day’s worth of energy) but around 40 times that amount in fat deposited inside of adipocytes (fat cells).

**Figure 9:**
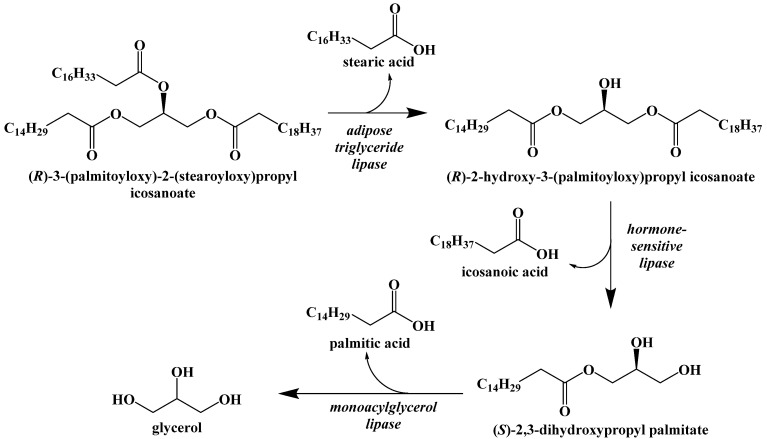
Lipolysis of Stored Triacylglycerides.

However, if glycogen stores are depleted and no carbohydrates are consumed, the body will begin to break down both dietary triacylglycerides and triacylglycerides contained in adipose cells through the process of lipolysis (Figure 9) [38]. The first step in lipolysis is the removal of the first fatty acid, in this case stearic acid, from the triglyceride. It requires a unique lipase called adipose triglyceride lipase. Although there are three diacylglycerol stereoisomers, adipose triglyceride lipase shows a preference for hydrolyzing the ester at the sn-2 position [39]. Similarly, hormone-sensitive lipase hydrolyzes diacylglycerol with a preference for 1,3-diacylglycerols. Monoacylglycerol lipase hydrolyzes the final fatty acid to generate glycerol and a net total of three fatty acids. These enzymes are relatively nonspecific because of the large variety of unique triacylglcerides in the diet.

Dietary fats are broken down by a similar method. Lingual lipases secreted in the mouth and gastric lipases in the stomach break down some triacylglycerides into mono- and diacylglycerides. Bile salts in the small intestine emulsify fats and allow pancreatic lipase to do the same. Free fatty acids and the mono- and diacylglycerides are then absorbed by intestinal mucosal cells and combined to form triacylglycerides that can be dissolved in lipoprotein complexes called chylomicrons. Chylomicrons are able to carry insoluble fats through the bloodstream. They are absorbed by liver, adipose, and muscle cells where the triacylglycerides are hydrolyzed by lipoprotein and hepatic triglyceride lipases to generate free fatty acids.

**Figure 10:**
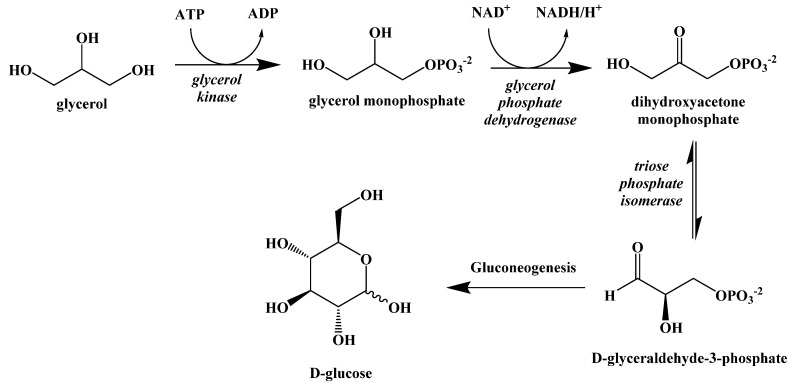
Metabolic Conversion of Glycerol to Glucose.

The fatty acids released either through lipolysis or through the digestion of dietary fats are carried through the blood by albumin until they are absorbed by cells and used as an energy source. The glycerol released by the breakdown of triacylglcyerides can be used to synthesize new triacylglycerides or can be converted in the liver to D-glyceraldehyde-3-phosphate and enter the gluconeogenesis pathway to generate glucose, but this will not fully meet the caloric requirements of the body (Figure 10).

**Figure 11:**
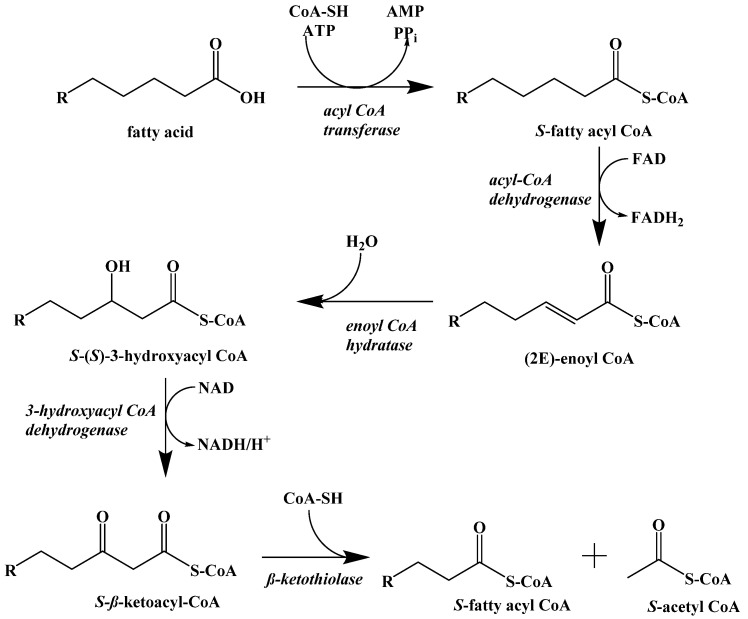
β-Oxidation of a Fatty Acid.

Instead, the fatty acids released by lipolysis are absorbed by the cells and converted into S-acetyl CoA through β-oxidation. This process is a repeating set of steps that converts the β-carbon of the fatty acid into a carbonyl group and then cleaves off an S-acetyl CoA, shortening the fatty acid (Figure 11).

The first step in fatty acid β-oxidation is the thioesterification by coenzyme A, which assists in transport to the mitochondrial outer membrane. Inside the mitochondrion, the first step is a regioselective dehydrogenation reaction. Next, the alkene is hydrated to generate a hydroxyl group at the β-carbon. This addition is stereoselective for the S-enantiomer. The penultimate step is the NAD^+^ oxidation of the secondary alcohol to a ketone. Finally, S-acetyl CoA is cleaved, and the resulting fatty acid chain is two carbon atoms shorter. The cycle repeats itself until the fatty acid is fully converted to S-acetyl CoA with some minor differences for unsaturated fatty acids and those with odd-numbered carbon chain lengths.

The S-acetyl CoA generated by cells through β-oxidation can directly enter the Krebs cycle and provide energy in the form of ATP. For most of the cells in the body, this process could supply their energy needs until triacylglyceride stores are depleted. However, because fatty acids cannot cross the blood–brain barrier, they cannot be absorbed and used by cells in the brain. The brain uses approximately 20% of the energy the body requires, which necessitates an effective method for providing lasting energy when carbohydrate consumption is low [3,40].

The body overcomes this obstacle by converting S-acetyl CoA into the three ketone bodies (Figure 1), water-soluble compounds that can enter the blood, cross the blood–brain barrier, and be absorbed by neuronal cells in the brain before being converted to S-acetyl CoA to fuel the Krebs cycle. While they are presented here in their acid forms, these two acids are deprotonated at physiological pH and referred to as acetoacetate and (R)-β-hydroxybutyrate [41].

**Figure 12:**
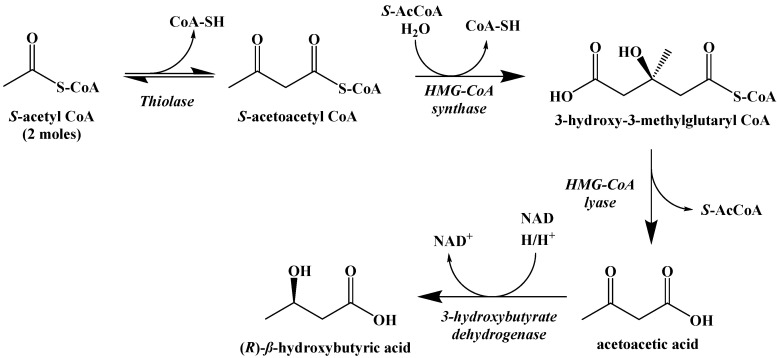
Ketogenesis of Ketone Bodies from S-Acetyl CoA.

The metabolic process of generating ketone bodies from S-acetyl CoA occurs through ketogenesis, and takes place primarily in the liver (Figure 12). The first step is the Claisen-type condensation of two molecules of S-acetyl CoA to form S-acetoacetyl CoA. This is followed by an aldol addition of another S-acetyl CoA to the β-carbonyl with subsequent hydrolysis of one of the coenzyme A thioesters to generate a carboxylic acid. The S-acetyl CoA is cleaved by HMG-CoA lyase to generate acetoacetic acid, which can subsequently be reduced by 3-hydroxybutyrate dehydrogenase to generate (R)-*β*-hydroxybutyric acid. Decarboxylation of acetoacetic acid, as a means to release acetone and carbon dioxide, can occur spontaneously as well, although this is primarily the method through which excess ketone bodies are removed as waste.

**Figure 13:**
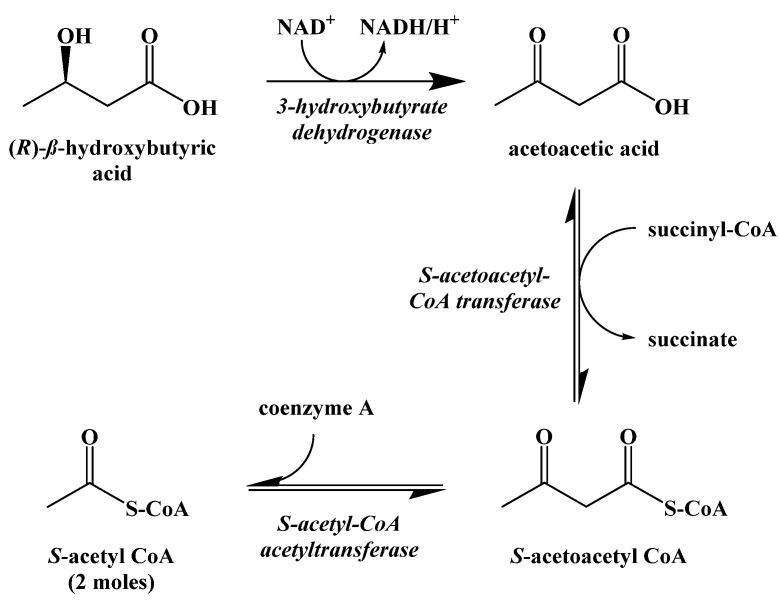
Ketolysis of the Ketone Bodies.

While S-acetoacetyl CoA could be hydrolyzed to acetoacetic acid directly, there is evidence that the expression of HMG-CoA synthase plays a role in limiting the rate at which ketogenesis occurs [42]. The ketone bodies produced metabolically in the liver are released into the blood where they can be absorbed by other cells and used to synthesize S-acetyl CoA through the process of ketolysis (Figure 13). First, NAD^+^ oxidation converts (R)-β-hydroxybutyric acid to acetoacetic acid, which undergoes thioesterification with coenzyme A to generate S-acetoacetyl CoA. S-Acetoacetyl CoA is cleaved by coenzyme A through a retro-Claisen reaction to afford two S-acetyl CoA molecules that can directly enter the Krebs cycle.

## 6. Metabolic Effect of Ketolysis Versus Glycolysis

Bypassing the traditional pathways of releasing energy through glycolysis in favor of using ketone bodies has a profound effect on the body. While the complete mechanism is not fully understood, bypassing carbohydrate metabolism pathways in the brain can lead to a decreased incidence or even the elimination of epileptic seizures [6].

The state of having elevated ketone bodies in the blood is called ketosis. Typical ketone body concentrations during dietary ketosis are between 0.5–3 mM, while a normal diet will give ketone body concentrations of less than 0.3 mM [43]. Achieving and keeping the body in a state of ketosis is the goal of the ketogenic diet, as any carbohydrates above an absolute bare minimum will trigger the release of insulin and rapidly decrease the rate of generation of ketone bodies.

It is important to note that this is different than diabetic ketoacidosis, a potentially life-threatening condition that occurs primarily in type I diabetics where unchecked ketogenesis can cause ketone body concentrations to rise to 10mM and beyond, overwhelming the body’s acid-base buffering system and causing the blood to turn acidic [44]. Insulin acts as an inhibitor for lipolysis, β-oxidation, and ketogenesis and prevents the build-up of ketone bodies to unsafe levels during dietary ketosis [43]. Even for those with type 2 diabetes where insulin sensitivity in the cells is decreased, diabetic ketoacidosis is rare since these catabolic processes are very sensitive to insulin. However, type I diabetics are at risk for diabetic ketoacidosis since the hormone to stop the generation of ketone bodies is present in low amounts or absent entirely.

## 7. Benefits of the Ketogenic Diet

The use of the ketogenic diet as a treatment option for epilepsy is well-studied, but research has shown that there are other benefits. The most popular is its efficacy as a weight-loss tool. Statistical analysis of fourteen studies comparing weight loss using the ketogenic diet or low-fat diets showed the ketogenic diet caused greater reductions in body weight [45]. The diet has also been shown to be a more effective method for weight loss compared to a normal diet at similar caloric deficits in trials that range in length from three months to two years [46,47]. It is possible that the initial rapid weight loss when initiating the diet comes from the body’s attempts to use glycerol from triglycerides to overcome a lack of dietary carbohydrates, leading to a decrease in adipose fat stores and thus weight. Converting glycerol into glucose through the gluconeogenesis pathway is also a very energy-demanding process, so the body is using excess energy before it begins to adapt to using ketone bodies as a primary energy source.

Tangentially related to the benefits of weight loss, the ketogenic diet has been shown to improve and even reverse insulin resistance in those suffering from type 2 diabetes or for those who are at risk of becoming diabetic [48,49]. Increased insulin resistance has been shown to lead to higher conversion of glucose into triglycerides that can lead to heart disease [50,51]. For obese patients, the ketogenic diet has been shown to decrease total cholesterol and triglyceride concentrations in the blood, which can decrease the risk of cardiovascular and metabolic diseases [52,53,54].

Alongside the more well-studied benefits of the diet, there is emerging evidence indicating benefits for other diseases and neurological disorders [55,56,57]. Patients with Parkinson’s disease have shown improved scores on the Unified Parkinson’s Disease Rating Scale after four weeks on a ketogenic diet [58]. The infusion of (R)-β-hydroxybutyric acid was shown to protect against dopaminergic neurodegeneration induced by neurotoxins that mimic the effects of Parkinson’s disease and Alzheimer’s disease [59,60]. Mouse models have been used to show that the ketogenic diet can decrease the concentration of amyloid-β in the brain, a risk factor for developing Alzheimer’s disease [61]. The ketogenic diet has also demonstrated in mice models to slow the degradation of motor neurons due to amyotrophic lateral sclerosis (ALS) [62].

It has long been recognized that cancer cells consume glucose at a much higher rate than normal cells [63,64]. Increased glycolysis promotes excessive proliferation in cancer cells [65]. However, in vitro studies have shown that a lack of glucose can cause apoptosis in malignant cells [66,67,68]. Certain tumors are unable to utilize ketone bodies as a source of energy due to a decrease in ketolysis enzyme activity [69,70]. The use of the ketogenic diet to effectively “starve” certain types of tumors could be an adjuvant treatment alongside more traditional forms of cancer treatment. In mice, the ketogenic diet has been shown to slow the rate of tumor growth and improve the effects of radiation treatment [71,72,73]. There are a few published reports of similar outcomes in human patients, but no formal clinical testing has yet been done [74,75]. While much of this evidence is far from conclusive, it shows the wide range of possibilities the diet holds, which justifies and indeed creates opportunities for further research.

## 8. Limitations of the Ketogenic Diet

Most of the long-term effects of the ketogenic diet are based on reports from the children with epilepsy who were treated by it. Hyperlipidemia has been seen in a majority of children treated with the traditional ketogenic diet, although this can be avoided by adjusting the types of fats consumed [76,77]. Kidney stones are seen in approximately 10% of children on the ketogenic diet [78,79]. There is concern that a strict ketogenic diet may affect growth rates, possibly due to protein or overall calorie restrictions [80,81]. There is some evidence that the ketogenic diet can cause osteopenia, a condition where bone mass is lost, possibly due to vitamin D and calcium deficiencies [82,83]. Research into the long-term effects of the ketogenic diet on healthy adults is sparse, but the recent popularity of the diet makes it likely that these studies will be done. 

One of the more common criticisms of the ketogenic diet is that it is difficult to maintain. Clinical studies from the diet’s earliest use in the 1920s to treat epilepsy and more recent studies on weight loss commonly report patients that were unable to follow the diet [20,21,22,23,45,46,47]. A single meal with too high a proportion of carbohydrates will cause the body to fall out of ketosis and revert back to using carbohydrates as its main energy source [84,85]. Long-term adherence to the diet can be low due to lack of discipline, adverse gastrointestinal effects, or palatability issues. 

Modified versions of the ketogenic diet have been developed to try and address these concerns. The MCT (medium-chain triglyceride) diet in particular has seen wide use. It focuses on using naturally-occurring triacylglycerides consisting of medium-chain fatty acids (between 6 and 12 carbons) that do not require active transport into cells and are instead absorbed by the liver directly. This increases the rate at which dietary triglycerides can be converted to ketone bodies, but research has shown that a greater percentage of fatty acids in medium-chain triglycerides are metabolized in the liver and can be used to generate ketone bodies compared to longer fatty acids that are partially used in the synthesis of new triacylglycerides to be stored in adipose tissue [86]. Medium-chain triglycerides have been shown to have a greater effect on ketone body concentration compared to long-chain triglycerides [87]. Because of this, the MCT diet allows for a greater amount of carbohydrates and proteins in the diet and medium-chain triglycerides are considered to be more “ketogenic”; however, it still suffers from many of the same drawbacks as the traditional ketogenic diet, including a reliance on a strict dietary regiment.

Improving the effectiveness and sustainability of the ketogenic diet using alternative natural sources of fat does not address many of the primary limiting factors of the diet. Preliminary research into the use of synthetic compounds to induce ketosis could better address these factors.

## 9. Synthetic Ketogenic Compounds as an Alternative Path to Ketosis

**Figure 14:**
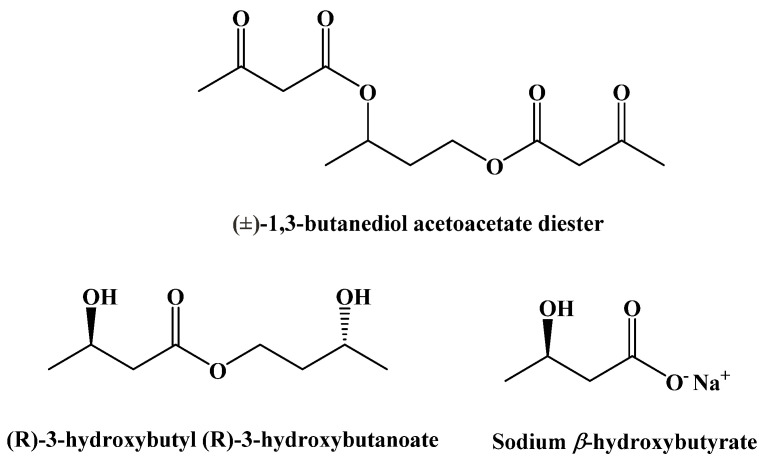
Synthetic Ketogenic Compounds.

**Figure 15:**
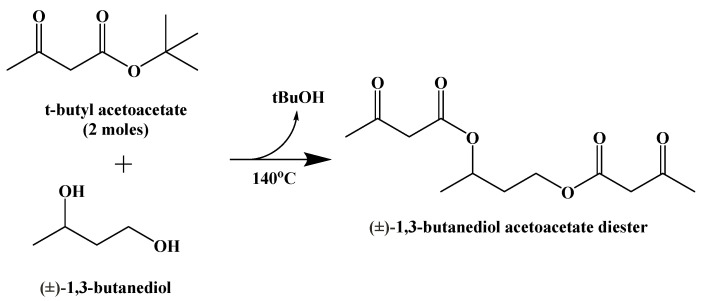
Synthesis of (±)-1,3-Butanediol Acetoacetate Diester.

Originally, it was believed that the benefits of the ketogenic diet were due to the decrease in glucose metabolism; however, more recent studies have shown that some of these benefits are instead caused by an increase in ketone body concentrations in the blood [88,89]. In the past, this distinction was irrelevant since a normal diet will prevent the body from generating ketone bodies at a rate high enough to maintain a state of ketosis. However, three synthetic ketogenic compounds have been shown to increase ketone body concentration in the blood to the point where dietary ketosis can be maintained without a change from a normal diet (Figure 14).

(±)-1,3-Butanediol acetoacetate diester was synthesized from (±)-1,3-butanediol and t-butyl acetoacetate via a transesterification, published in 1995 (Figure 15) [90].

**Figure 16:**
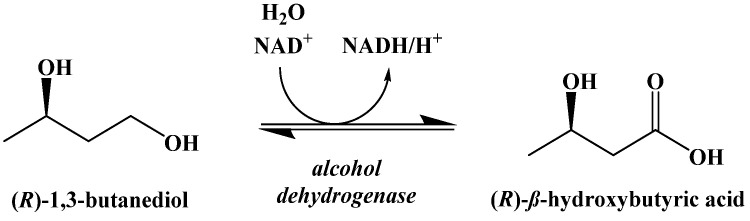
Oxidation of (R)-β-1,3-Butanediol in the Liver.

This compound is metabolically hydrolyzed to generate two equivalents of acetoacetic acid and (±)-1,3-butanediol, which can be oxidized to generate β-hydroxybutyric acid (Figure 16) [91,92]. The oxidation of the (R)-enantiomer occurs in the liver. (S)-β-Hydroxybutyric acid is not naturally occurring, but its metabolism in perfused rat livers has been shown to generate ketone bodies through an unknown pathway [93].

Racemic 1,3-butanediol acetoacetate diester was shown to increase ketone body concentrations in the blood of both a pig and dog. More recent studies in rats indicate it induces a rapid increase in (R)-β-hydroxybutyric acid concentration in the blood and a decrease in blood glucose concentration, indicative of ketosis [94]. Importantly, triglyceride and cholesterol levels did not change to a statistically relevant level over 28 days of daily administration [6].

**Figure 17:**
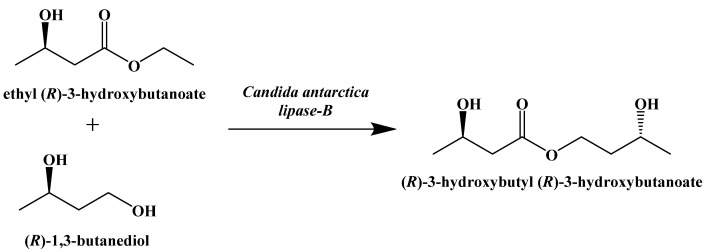
Enzymatic Synthesis of (R)-3-hydroxybutyl (R)-3-hydroxybutanoate.

A similar compound, (R)-3-hydroxybutyl (R)-3-hydroxybutanoate, was synthesized by Clarke and Veech in 2010 [95]. A lipase-mediated transesterification reaction between (R)-1,3-butanediol and ethyl (R)-3-hydroxybutanoate generates a stereospecific monoester that can be hydrolyzed to generate (R)-β-hydroxybutyric acid and (R)-1,3-butanediol, which can be oxidized in the liver to give a second equivalent of (R)-β-hydroxybutyric acid (Figure 17) [96].

It has been shown in adult humans that daily administration of (R)-3-hydroxybutyl (R)-3-hydroxybutanoate can safely induce ketosis while on a normal diet over 28 days of administration [97,98].

**Figure 18:**
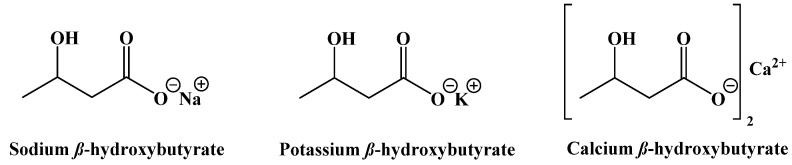
β-Hydroxybutyrate Mineral Salts.

A final set of ketogenic compounds that have been used to induce ketosis are salts generated from racemic β-hydroxybutyrate. Most commonly, these are sodium, potassium, and calcium salts (Figure 18). In studies in rats fed a normal diet and supplemented with these salts, a small increase in ketone body concentration in the blood was observed. Mineral salts of β-hydroxybutyrate can induce ketosis, but the amounts required can lead to negative gastrointestinal effects and issues due to high levels of sodium [99].

The use of synthetic ketogenic compounds to supplement a normal diet has not only shown the capability to induce ketosis, but also consequently decreases blood glucose concentration without affecting triglyceride or cholesterol levels [6]. (±)-1,3-Butanediol acetoacetate diester has been shown to suppress seizure activity in rats [100]. Combining (±)-1,3-butanediol acetoacetate diester with hyperbaric oxygen therapy was shown to slow metastatic cancer growth in mice [5]. Like the benefits of the ketogenic diet itself, the research into the use of synthetic ketogenic compounds to supplement a normal diet is promising but is in its infancy. Similarly, the variety of synthetic ketogenic compounds available is currently extremely limited to those described, and thus there remains enormous potential to further expand on this through targeted organic synthesis, to advance the breadth of research on ketogenic substances and their use in improving human health.

## 10. Conclusions

From a chemist’s perspective, the field of synthetic ketogenic compounds is vast and virtually unexplored. Palatability, caloric density, solubility, and gastrointestinal tolerance are all attributes that can be improved. Advances in food formulation of lipid emulsions may be the most obvious immediate options, but there is also a need for a significant expansion of edible, ketogenic food substances whose structures, purities, and biochemical effects are all well-documented. Different functional groups linking together ketogenic groups could lead to a better controlled rate of generation of ketone bodies, perhaps even tissue-specific. The possibilities are nearly endless and limited only by the imaginative abilities and restrictions of chemical synthesis.

It is rare to find an area of research in the field of chemistry that is mostly unexplored. Small-molecule synthesis is of particular interest to organic chemists for pharmaceutical development across a broad spectrum of human diseases, but whether it is the ketogenic diet’s origins as a medical treatment or the relative recency of much of the research, there has been little effort put forth to explore the field of ketogenic compounds among synthetic organic chemists. There is both commercial and biomedical potential to providing alternative methods of inducing and maintaining ketosis, and novel synthetic ketogenic compounds could be an important aspect of a solution.
